# Online Sepsis Prediction Using Vital Signs and Multiscale Temporal-Aware Contrastive Learning: Model Development and Validation Study

**DOI:** 10.2196/82762

**Published:** 2026-06-19

**Authors:** Xiaoqiong Yang, Zezhong Lv, Hanming Lv, Qianyi Zhou, Wei Jiang, Ziqun Meng, Wenjie Yang

**Affiliations:** 1 Department of Infectious Diseases, Tianjin First Central Hospital, Baoshan West Road, 2nd, Tianjin, 300190, China, (86) 13602155376; 2 School of Control Science and Engineering, Tiangong University, Tianjin, China; 3 School of Textile Science and Engineering, Tiangong University, Tianjin, China; 4 Clinical Medical College, Tianjin Medical University, Tianjin, China

**Keywords:** sepsis prediction, deep learning, vital signs monitoring, temporal modeling, contrastive learning

## Abstract

**Background:**

Real-time prediction of sepsis is a critical yet highly challenging task. Existing studies face 2 major limitations. First, they often rely on laboratory test results that are not readily available in real time, making timely diagnosis difficult. Second, the patient’s condition evolves as a typical time series, but current methods often adopt coarse modeling strategies, with model architectures that are inefficient to train and deploy effectively.

**Objective:**

This study aimed to develop a prediction model for online sepsis detection using only easily obtainable vital signs, such as heart rate and temperature, with variable-length input sequences while maintaining high predictive performance through the multiscale temporal representation learning.

**Methods:**

We propose a deep learning model, Multi-Scale Temporal-aware Contrastive Learning (MSTCL), for efficient sepsis prediction based on the intensive care unit data derived from publicly available databases. We propose a multiscale temporal model to capture both short- and long-term dependencies in variable-length physiological time series. To enhance the robustness of our model, we used contrastive learning techniques that differentiate between positive and negative sepsis progression trajectories. Input features were limited to 6 vital signs, without reliance on laboratory tests or clinical notes.

**Results:**

The model was evaluated on more than 400 patients with and without sepsis. It achieved an area under the receiver operating characteristic curve of 88.34%, a sensitivity of 89.29%, and a specificity of 73% for predicting sepsis onset based on variable-length vital-sign histories. The normalized mean absolute error for the predicted sepsis onset was 0.11%.

**Conclusions:**

Our proposed model’s low complexity and rapid inference make it suitable for deployment in real-time monitoring systems and low-resource environments. The ability to learn from variable-length historical data enhances the clinical applicability of our model. Furthermore, the methodology of temporal-aware contrastive learning offers a robust and efficient solution for online sepsis detection in diverse clinical settings.

## Introduction

Sepsis is a life-threatening clinical syndrome caused by a dysregulated host response to infection, leading to acute organ dysfunction [1]. It is a major global health concern, accounting for an estimated 270,000 deaths annually, nearly 30% of all hospital deaths [2,3]. Timely identification and early intervention are critical for improving patient outcomes, as delays in treatment significantly increase mortality and morbidity [4,5]. Accordingly, the development of accurate and timely predictive models for sepsis onset remains a high-priority task in clinical informatics, with the potential to substantially reduce adverse outcomes through early clinical alerts and proactive management.

Traditional approaches to sepsis prediction, such as the Sequential Organ Failure Assessment and quick Sequential Organ Failure Assessment scores [6], are widely used in clinical practice. These scoring systems rely on predefined thresholds and combinations of physiological and laboratory measurements to assess patient risk. However, their performance is limited by relatively low sensitivity, especially in the early stages of sepsis [7]. Moreover, their reliance on laboratory-based values restricts their applicability in settings where such information is delayed or unavailable [8]. As a result, there has been growing interest in leveraging machine learning and, more recently, deep learning approaches to improve predictive performance in sepsis detection.

Deep learning methods have shown promising results in sepsis prediction tasks [5,9], particularly models based on recurrent neural networks (eg, long short-term memory [LSTM] networks) and convolutional neural networks (CNNs). These models can capture temporal patterns and nonlinear dependencies in high-dimensional physiological data. For example, the Smart Sepsis Predictor [10], using an LSTM-CNN-fully connected architecture trained on intensive care unit (ICU) data, achieved an area under the receiver operating characteristic curve (AUROC) of up to 0.89 using only demographic and vital sign features, and up to 0.92 when laboratory data were included. Another large-scale study [11] used only 5 vital signs and patient age to predict sepsis mortality within 6 to 48 hours, reporting areas under the curve (AUCs) of 0.84 for CNN and 0.761 for LSTM for a 6-hour lead time. Though promising, many of these approaches suffer from practical limitations: they often require a large number of input features, including laboratory results, demographics, and textual data, which limits their deployment in real-time or low-resource settings. Additionally, many models assume fixed-length input sequences, which is incongruent with the inherently irregular and variable-length nature of clinical time-series data. These constraints hinder the usability and generalizability of existing methods across diverse clinical environments.

Recently, transformer-based models [12] have emerged as powerful alternatives for sequence modeling tasks. Originally designed for natural language processing, transformers excel in capturing temporal dependencies through self-attention mechanisms, without relying on recurrent structures. The ability to model sequential data makes transformers particularly well-suited to clinical applications, where patient data may evolve at varying rhythms and durations. For instance, Tang et al [13] proposed CNN-transformer and LSTM-transformer hybrid models for early sepsis prediction, demonstrating nearly 20% improvement over baseline recurrent neural network models for 4- to 12-hour preonset windows. Another recent study by Chang et al [14] designed a transformer-based diffusion probabilistic model to forecast vital signs (heart rate, systolic blood pressure, and diastolic blood pressure) in ICU patients, achieving substantial gains in inference speed over baselines using MIMIC-III data [15]. However, existing transformer-based approaches often still rely on relatively rich sets of inputs—including laboratory measurements and demographic features—or presume that sufficient historical data are available.

In this work, we harnessed the strengths of the transformer architecture to build a lightweight, flexible, and high-performing model for real-time sepsis prediction. Specifically, we proposed Multi-Scale Temporal-aware Contrastive Learning (MSTCL), which models the temporal dependencies in routinely monitored vital signs. Unlike conventional approaches that require a fixed window of input data, our model supports variable-length sequences and enables efficient online inference through an autoregressive encoder-decoder design. This allows real-time prediction of sepsis onset using only a short and dynamically updating history of patient monitoring data, making it suitable for real-time warning systems in intensive care and emergency settings.

To further enhance the model’s robustness and applicability in diverse clinical conditions, we intentionally restricted the input features to 6 easily obtainable and commonly monitored vital signs: oxygen saturation, heart rate, body temperature, systolic blood pressure, diastolic blood pressure, and respiratory rate. While this focus on noninvasive data distinguishes our work from methods relying on exhaustive laboratory results, it introduces a significant technical challenge: the reduction in input features may limit the model’s ability to capture the subtle, high-dimensional physiological precursors of sepsis. To bridge this information gap and ensure high predictive performance despite the simplified inputs, we proposed the MSTCL framework.

Specifically, in this study, we proposed a hybrid contrastive learning objective that distinguishes patient vital sign sequences from 3 perspectives: positive segments, negative segments, and full sequences, with the last serving as transitional intermediaries. By enforcing distinct distances among the representations of these 3 types of sequences, the model achieves more stable perception of complex dependencies within patient data streams.

In summary, the proposed framework enables multiscale temporal modeling and contrastive representation learning in a lightweight, autoregressive architecture, consuming only 6 vital signs as input. This enables real-time, variable-length sepsis prediction across diverse clinical environments. Our approach demonstrates that accurate online prediction of sepsis is feasible using easily obtainable physiological data while maintaining robust generalization and operational efficiency.

## Methods

### Dataset

The training and testing data used in this study were sampled from the publicly available PhysioNet Computing in Cardiology 2019 Challenge dataset [16]. This dataset includes ICU records from patients in 3 different hospitals, of which data from 2 hospitals are openly accessible. These 2 sources provide a total of 40,336 patient records, each containing hourly time-stamped information on demographics, vital signs, and laboratory values, which is referred to in this paper as the “original dataset.” However, most of these records are temporally sparse. In many cases, even over dozens of recorded hours, most laboratory values and some vital signs had only a few valid entries. This sparsity highlights the difficulty of obtaining real-time laboratory values in most clinical settings, which in turn motivates our study: to enable real-time sepsis prediction using only easily monitored and frequently available vital signs. To achieve this, we applied the following data filtering procedure. First, we separated the original dataset into records of patients with sepsis (with confirmed sepsis onset) and patients without sepsis (entirely negative throughout the ICU stay). For nonseptic records, we randomly sampled sequences in which the missing rate of vital signs was no more than 50%. For septic records, we first identified the sepsis onset point and then examined a 16-hour window surrounding it. If the missing rate of the 6 selected vital signs within that window was less than 50%, the record was retained in our dataset. This selection was based on the assumption that, to train a real-time predictive model—especially when using a limited number of input variables—the training data must exhibit reasonably high temporal quality. Only with sufficient continuity and density in the input signals can the model effectively learn and capture the underlying temporal dependencies necessary for accurate sepsis onset prediction. Table 1 summarizes the overall demographics of the data sampled from the source dataset for training and testing in this study. The number of qualified vital sign sequences from positive and negative patients was 1093 and 2673, respectively, totaling 3766 sequences—less than one-tenth of the original dataset size of 40,336. The sex ratio and age distribution of the sampled dataset were generally consistent with those of the original dataset. For both positive and negative patients, the number of samples increased progressively with age. The age distribution of positive patients exhibited a more pronounced long-tail pattern compared with that of negative patients. Due to the temporal sparsity of the original PhysioNet Challenge data, we trained and evaluated the proposed MSTCL framework based on these processed samples, which were split into training and test sets using a 4:1 ratio.

**Table 1.** Demographic characteristics of patients with sepsis and patients without sepsis.

Demographic variables	Septic (n=1093), n (%)	Nonseptic (n=2673), n (%)	Overall (n=3766), n (%)	Original data (n=40,336) [16], n (%)
Male	656 (60)	1823 (68.2)	2478 (65.8)	22,568 (56)
Age group (years)				
0‐24	29 (2.7)	19 (0.7)	48 (1.3)	972 (2.4)
25‐39	76 (7)	87 (3.3)	163 (4.3)	3251 (8.1)
40‐54	204 (18.7)	420 (15.7)	625 (16.6)	8108 (20.1)
55‐64	232 (21.2)	671 (25.1)	904 (24)	8633 (21.4)
≥65	552 (50.5)	1476 (55.2)	2026 (53.8)	19,372 (48)

### Model Architecture

**Figure 1.**
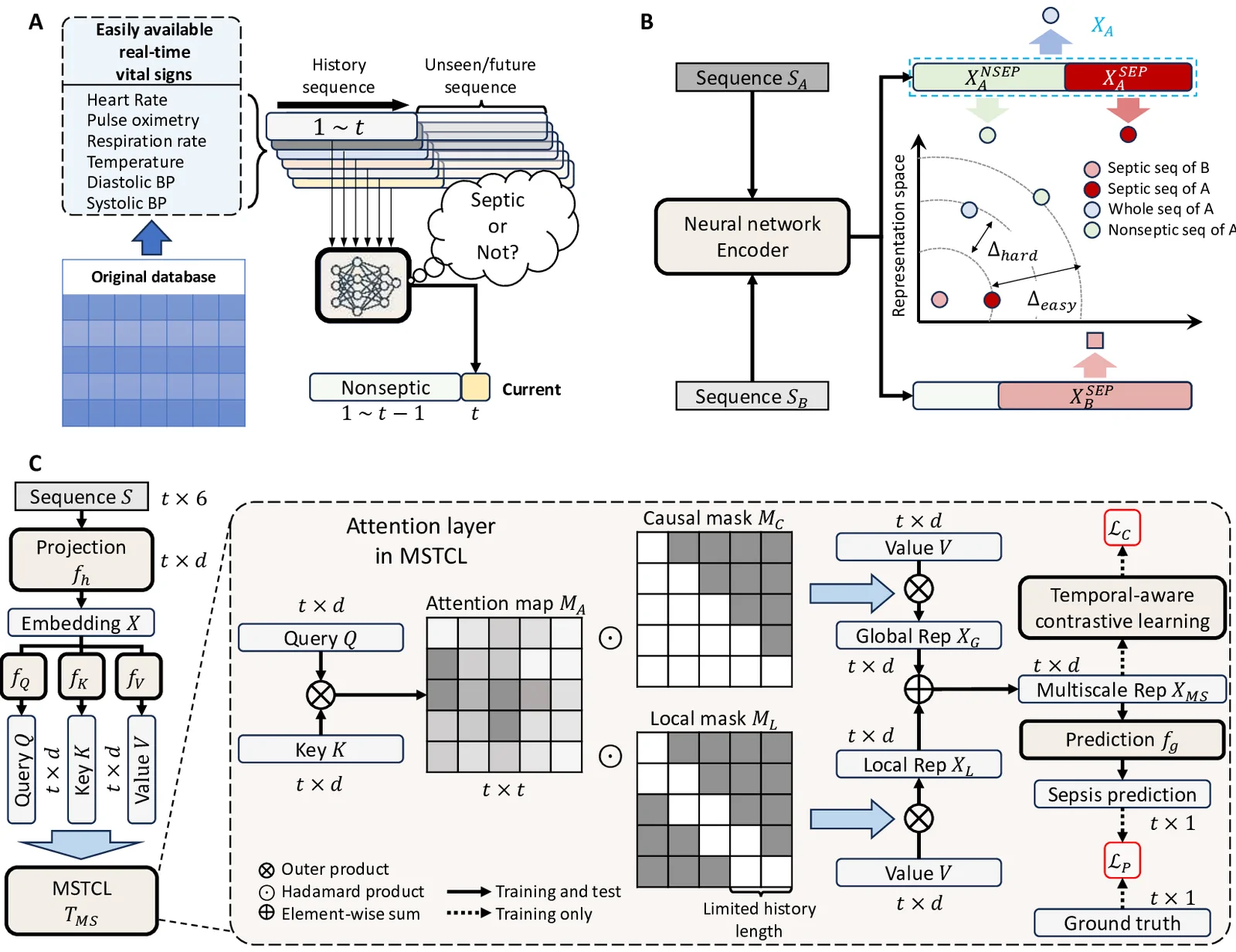
Overview of the proposed method: (A) The methodology enables online sepsis prediction using only real-time, easily accessible vital signs. (B) Hybrid contrastive learning framework. (C) The autoregressive Multi-Scale Temporal-aware Contrastive Learning (MSTCL) architecture using multiscale causal masks, where darker colors represent lower values.

Figure 1 presents an overview of our proposed MSTCL model. We focus on real-time sepsis prediction using only 6 easily obtainable vital signs: oxygen saturation, heart rate, body temperature, systolic blood pressure, diastolic blood pressure, and respiratory rate. As illustrated in Figure 1A, we collected high-quality, continuous sequences from the original dataset to construct our training and testing datasets. The goal of this study was to design a neural network model capable of predicting the current likelihood of sepsis onset in real time, based on variable-length sequences of these vital signs. To effectively model the relationship between this limited set of vital signs and sepsis onset, we proposed a multiscale temporal dependency modeling architecture, MSTCL ($T_{MS}$), as presented in Figure 1C, which is equipped with the contrastive learning based on the vital sign sequence representations shown in Figure 1B.

Specifically, during training, a given vital sign sequence $S\in R^{t\times 6}$ is first embedded into $X\in R^{t\times d}$ via a linear layer, $X=f_{h}\left(S\right)$, where t is the temporal length of the 6D vital signs and *d* is the hidden dimension of the model. Then, the projections for query, key, and value are computed to obtain $Q,\;K,\;V\in R^{t\times d}$, respectively. The attention map $M_{A}\in R^{t\times t}$ is computed as the matrix product between Q and K.

To simulate sepsis prediction based solely on the historical vital signs during test time, a causal mask $M_{C}\in R^{t\times t}$ is applied during training. This is achieved via a Hadamard product between $M_{A}$ and $M_{C}$, ensuring that the attention mechanism is restricted to time steps prior to the current one (ranging from 1 to *t*), resulting in a lower-triangular matrix as illustrated in Figure 1C. The global autoencoded representation of the input sequence is then obtained by the following methodology with residual connections and activation functions omitted for clarity:



(1)
$$X_{G}=Q^{T}⊗K⊙M_{C}⊗V,X_{G}\in R^{t\times d}$$

While the transformer was originally proposed to model global dependencies in sequence data, sepsis onset is often correlated with local temporal patterns. Therefore, we introduce a local self-attention branch equipped with a local mask $M_{L}\in R^{t\times t}$ to capture short-term dependencies. To implement this, earlier history beyond a predefined window is masked, such that each time step can only attend to a limited portion of the past, enabling localized temporal modeling. The local representation is obtained in a similar manner described in equation 1 as $X_{L}\in R^{t\times d}$. Finally, the multiscale representation of the vital sign sequence is computed as element-wise addition:



(2)
$$X_{MS}=T_{MS}\left(S\right)=X_{G}⊕X_{L}$$

Based on this multiscale representation, we proposed a contrastive learning framework to help the model discriminate between septic and nonseptic sequences. Specifically, as shown in Figure 1B, for any 2 given septic sequences $S_{A}$ and $S_{B}$, we extract the septic and nonseptic subsequence representations $X_{A}^{SEP}$ and $X_{A}^{NSEP}$ from $S_{A}$, along with its full-sequence representation $X_{A}$, and the septic subsequence representation$X_{B}^{SEP}$ from $S_{B}$. These representations are obtained by performing average pooling on the feature sequences encoded by the proposed MSTCL model (equation 2). For clarity, we omit the subscript MS on these representations.

Intuitively, the distance between $X_{B}^{SEP}$ and $X_{A}^{SEP}$ should be smaller than that between $X_{B}^{SEP}$ and $X_{A}^{NSEP}$, while the distance between $X_{B}^{SEP}$ and $X_{A}$ should lie in between. This assumption is visualized in the schematic of the representation space in Figure 1B. We formalize this assumption as a temporal-aware contrastive learning objective and incorporate it into the training process.

Finally, the sepsis prediction is performed using a linear layer $f_{g}$ followed by a sigmoid activation function to produce the probability of sepsis onset at each time step.



(3)
$$\hat{y}=f_{g}\left(X_{MS}\right),\hat{y}\in R^{t\times 1}$$

### Model Training and Test

The training objective of our model consists of 2 main components: a classification loss between the step-by-step sepsis onset predictions (positive or negative) and ground truth labels $y\in R^{t\times 1}$, simulated using a causal mask



(4)
$$L_{p}=-\frac{1}{t}\sum_{i=1}^{t}\left(y_{i}\log\left({\hat{y}}_{i}\right)+\left(1-y_{i}\right)\log\left(1-{\hat{y}}_{i}\right)\right),$$

and a contrastive learning loss based on multiscale representations of the vital sign sequences



(5)
$$\begin{array}{rl} L_{c}=\; & \max(0,\;d\left(X_{B}^{SEP},X_{A}^{NSEP}\right)-d\left(X_{B}^{SEP},X_{A}^{SEP}\right)+{△}_{easy})\\ +\max(0,\;d\left(X_{B}^{SEP},X_{A}\right)-d\left(X_{B}^{SEP},X_{A}^{NSEP}\right)+{△}_{hard}). \end{array}$$

where ${△}_{easy}=0.25$*,* ${△}_{hard}=0.2$ are set in this study by evaluating three configurations (0.2/0.15, 0.25/0.2, and 0.3/0.25), among which the setting of 0.25/0.2 yields the lowest mean absolute error (MAE) value of 0.113; d(⋅,⋅) denotes a distance measure, which is Euclidean distance based on L2-normalized features in this study. Our proposed MSTCL model optimized a combined loss that integrates both objectives.



(6)
$$L=L_{P}+L_{C}$$

The model was implemented using Python (Python Software Foundation) and PyTorch (version 2.1; PyTorch Foundation) and was trained with the Adam optimizer [17] with an initial learning rate of 1 × 10^–4^ for 100 epochs. The proposed MSTCL model contains approximately 0.6 million parameters. Compared with several existing deep learning–based methods, such as temporal convolutional networks [18] (approximately 0.4 million) and the method described by Li et al [19] (approximately 0.45 million), our approach achieved superior performance, even in an online prediction setting, while maintaining a comparable model size. During inference, as illustrated in Figure 1C, neither of the 2 training losses was required. The model is capable of predicting the likelihood of sepsis onset in real-time, based solely on the newly incoming vital sign values at each time step. The inference latency of our MSTCL was 19 milliseconds with 20 MB VRAM on NVIDIA GTX 1650 Ti (4 GB).

### Evaluation Metric

In the testing phase, the model encoded the incoming vital signs sequence and performed step-by-step online prediction of $\hat{y}$. When the model predicted a positive case of sepsis, the current sequence is marked as septic, and the difference between the predicted onset and the ground truth is measured using MAE normalized by the input sequence length. If the model does not predict any positive outcome throughout the entire test sequence, the sequence is classified as nonseptic. Accordingly, in addition to MAE, we also used AUROC, area under the precision-recall curve (AUPRC), sensitivity, and specificity to evaluate the model’s ability to distinguish between septic and nonseptic sequences. AUROC measures the trade-off between true positive and false positive rates across thresholds. AUPRC focuses on performance under class imbalance by evaluating precision and recall. Sensitivity (true positive rate) assesses the proportion of actual septic cases correctly identified, while specificity (true negative rate) reflects how well the model excludes nonseptic cases.

### Ethical Considerations

The publicly available, fully anonymized PhysioNet Computing in Cardiology 2019 Challenge dataset was used in this study. Our study protocol was approved by the Ethics Committee of Tianjin First Central Hospital, which waived ethical review and informed consent, and all experimental practices conformed to institutional and national human research ethics criteria and the World Medical Association Declaration of Helsinki.

## Results

Table 2 presents the performance of the proposed method in predicting sepsis onset, measured by AUROC, AUPRC, MAE, sensitivity, and specificity. The reported MAE values are normalized by the input sequence length.

Specifically, 2 types of temporal modeling strategies were considered: local and global, corresponding to “Loc” and “Glb” in the table. For negative sampling in contrastive learning, we compared 2 types: vital sign segments from only negative patients (NegSeq in Table 2), and full-length vital sign sequences (WholeSeq in Table 2). These served as “easy” and “hard” negatives, respectively, relative to the positive sequences derived from patients with sepsis. By performing ablations on these 4 components (eg, Loc, Glb, NegSeq, and WholeSeq), we assessed the individual contributions of each design choice. From the first and second rows of Table 2, we observed that even when negative sampling was optimized for contrastive learning, the absence of either local or global temporal modeling substantially degraded performance (eg, AUROC decreased by 22.9 and 20.9 points, respectively, relative to the best-performing configuration). This finding highlights the foundational role of capturing multiscale temporal dependencies in patient vital signs for effective sepsis prediction. Rows 3, 4, and 5 further examine the impact of introducing full-length sequences as harder, transitional negatives. These helped the model learn more discriminative representations by pushing it to distinguish subtle differences in complex temporal patterns. This effect is further demonstrated through visualization analysis.

**Table 2.** Effect of main components on the sepsis prediction performance. The results are obtained via the average across 5-fold cross validation.

	AUROC^a^, mean (SD)	AUPRC^b^, mean (SD)	MAE^c^, mean (SD)	Sensitivity, mean (SD)	Specificity, mean (SD)
Loc^d^ + NegSeq^e^ + WholeSeq^f^	65.39 (0.22)	63.16 (0.25)	0.33 (0.01)	61.10 (0.04)	59.11 (0.04)
Glb^g^ + NegSeq + WholeSeq	67.93 (0.22)	65.69 (0.25)	0.31 (0.01)	62.24 (0.04)	60.92 (0.04)
Loc + Glb	81.34 (0.19)	81.19 (0.22)	0.15 (0.00)	75.42 (0.02)	70.03 (0.02)
Loc + Glb + NegSeq	83.01 (0.18)	82.29 (0.21)	0.12 (0.00)	80.50 (0.02)	70.26 (0.02)
Loc + Glb + NegSeq + WholeSeq	*88.34 (0.13)^h^*	*86.74 (0.19)*	*0.11 (0.00)*	*89.29 (0.01)*	*73.02 (0.02)*

a AUROC: area under the receiver operating characteristic curve.

b AUPRC: area under the precision-recall curve.

c MAE: mean absolute error.

d Loc: local temporal modeling.

e NegSeq: vital sign segments from only negative patients.

f WholeSeq: full-length vital sign sequences.

g Glb: global temporal modeling.

h The best results are italicized.

**Figure 2.**
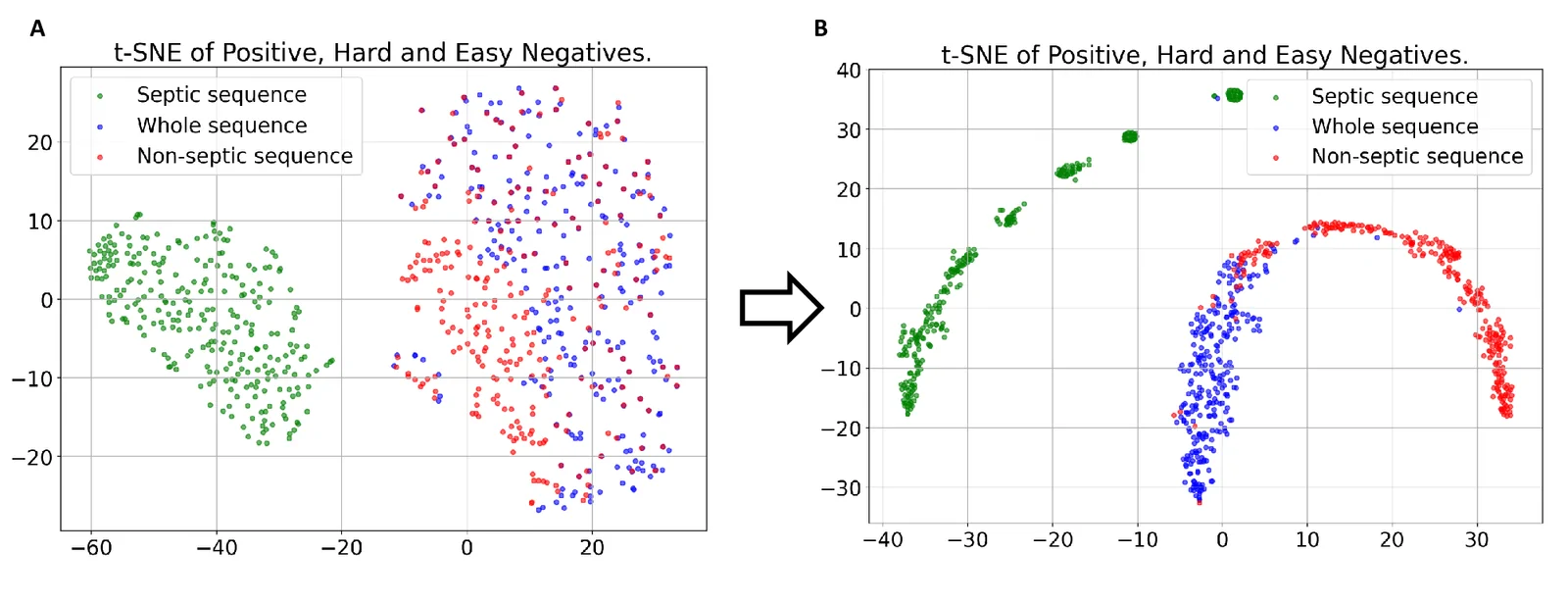
t-distributed stochastic neighbor embedding (t-SNE) visualization of vital sign representations before and after training. (A) Sequence representation before training and (B) sequence representation after training.

We applied the t-distributed stochastic neighbor embedding method to visualize the latent representations of patients’ temporal vital sign data before and after training. As shown in Figure 2, compared with the pretraining state (Figure 2A), the full-length vital sign sequences, which include both positive and negative segments, became more distinguishable from purely negative sequences after training (Figure 2B). This aligns with the natural assumption that full sequences are more similar to positive sequences than to negative sequences. Overall, the combination of multiscale temporal modeling and hierarchical contrastive learning yielded the best predictive performance across all metrics.

**Figure 3.**
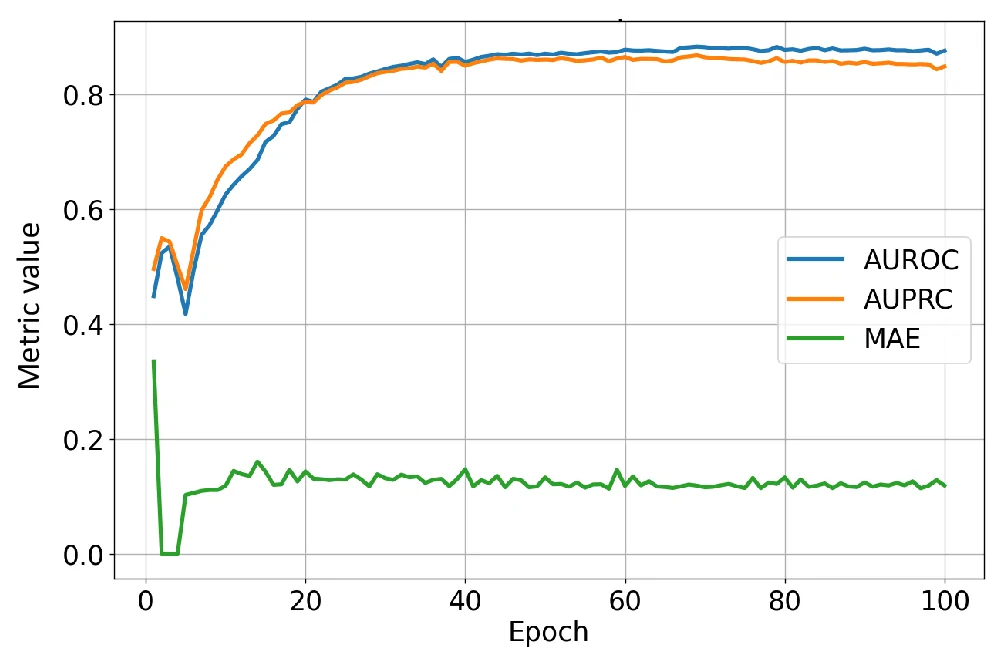
Visualization of model performance metrics across the training process. AUPRC: area under the precision-recall curve; AUROC: area under the receiver operating characteristic curve; MAE: mean absolute error.

Figure 3 illustrates the stabilization of AUROC, AUPRC, and MAE on the test dataset as the number of training epochs increased. The results cover the first 100 training epochs. All 3 metrics exhibited some fluctuation during the initial 10 epochs. After the 10th epoch, AUROC and AUPRC steadily improved, reaching their peak values, which were 88.3 and 86.7, respectively, around epoch 60, after which they plateaued. Meanwhile, the normalized MAE showed a gradual decline following the 10th epoch, with slightly more fluctuation than AUROC and AUPRC, indicating progressively more accurate predictions of sepsis onset timing. MAE also reached its lowest point around epoch 60, with greater stability in the later training stages compared with earlier ones. One possible reason for the relative instability of MAE is that it is a discrete metric, reflecting the difference between the predicted transition-to-positive point within the model’s output sequence and the actual onset time of sepsis.

**Figure 4.**
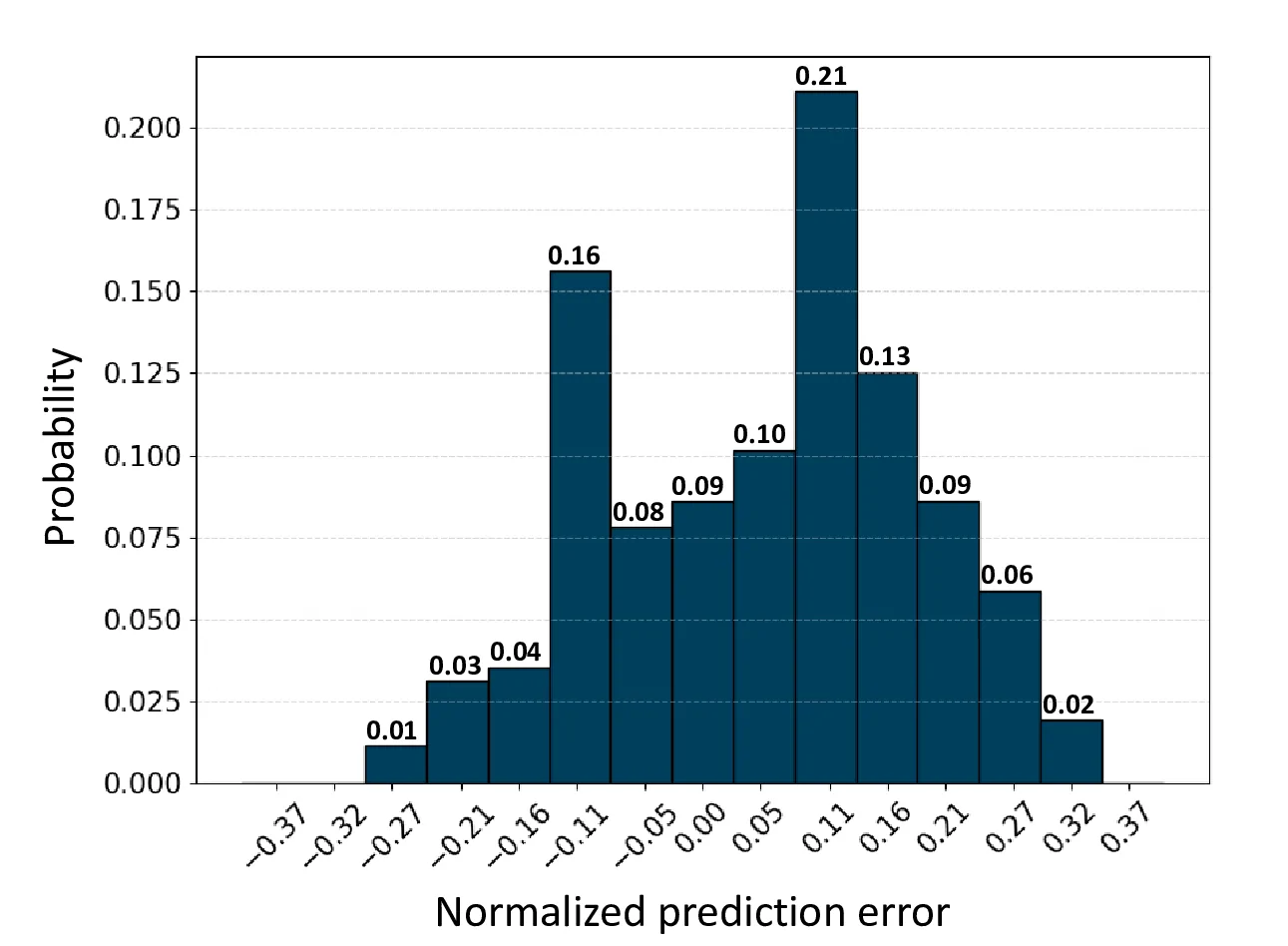
Distribution of sepsis prediction errors. Negative errors indicate early predictions before sepsis onset.

Figure 4 shows the probability distribution of the normalized prediction error, including the probabilities of correct, early, and delayed predictions among all detected sepsis sample sequences. It can be observed that the most frequent errors occur at a lead of 0.11 and a lag of 0.11, accounting for approximately 16% and 21%, respectively. Predictions that are approximately correct (±0.05) each account for about 8%, indicating that more than 64% of predictions fall within a ±0.11 error margin.

Table 3 compares the proposed method with existing related works across several key dimensions, including temporal modeling approach (temporal model), task type (manner), the number of training and testing samples used (samples), the number of input vital signs (input), required historical sequence length (history length), and performance metrics such as AUC and MAE. Regarding the evaluation metrics, we introduced MAE specifically to quantify the model’s ability to precisely locate the sepsis transition point in a continuous time series. While traditional metrics like AUC evaluate classification accuracy, MAE provides insight into the ‘early warning’ lead time, which is a unique focus of our MSTCL framework compared with existing single-point prediction methods. Among all methods listed, aside from our proposed approach, the kinematics approach with neural networks for early detection of sepsis (KANNEDS) [20] uses the fewest types of input signals. It is based on an LSTM architecture designed for sepsis identification but lacks the capability for real-time onset prediction and requires fixed-length historical sequences. The temporal convolutional network [18], on the other hand, supports variable-length input sequences for identifying patients with sepsis but relies on as many as 40 types of input signals and uses the entire original dataset. Zhao et al [21] proposed a model based on light gradient boosting machine, achieving the highest AUC among identification tasks, but it still requires fixed-length historical sequences as input. Li et al [19] introduced a powerful LSTM+CNN-based model capable of predicting sepsis onset, reaching an AUC of 0.96; however, it requires a large amount of training data to achieve such performance. In contrast, our proposed MSTCL achieves efficient and lightweight online sepsis prediction using only a small set of vital signs and approximately one-tenth of the data required by other methods. This demonstrates the model’s strong performance and generalizability even under limited data conditions.

To further investigate the importance of the selected 6 vital signs, we evaluated the model’s sensitivity by systematically masking each input signal (setting its value to zero) during the inference phase and observing the degradation in predictive performance compared with the full-feature model (ALL).

As verified by the MAE metrics obtained via leave-one-feature-out ablation over 6 vital sign inputs, excluding body temperature yielded the highest MAE (0.18) and triggered the most substantial performance decline, highlighting its critical role in identifying the systemic inflammatory response in sepsis. Removing oxygen saturation, heart rate, and systolic blood pressure separately led to moderate MAE increments of 0.14, 0.15, and 0.13, respectively, while eliminating either diastolic blood pressure or respiratory rate only caused a slight MAE elevation to 0.12; by contrast, incorporating all 6 vital signs achieved the most minimal MAE (0.11). Collectively, these results empirically support the clinical relevance of our 6-feature configuration, which strikes an effective balance between model complexity and predictive power.

The choice of margins ${△}_{easy}$ and ${△}_{hard}$ is crucial for hierarchical feature learning. The configuration of (0.25, 0.2) achieved the best prediction performance. However, the performance did not vary significantly with the changes in this parameter. Consequently, when applying the proposed MSTCL framework to other datasets, the setting of this parameter is unlikely to become a performance bottleneck.

**Table 3.** Comparison of the proposed model with several related approaches based on the PhysioNet Computing in Cardiology 2019 Challenge dataset.

Methods	Temporal model	Manner	Samples, n	Input data dimension	History length	AUC^a^	MAE^b^
Kinematics approach with neural networks for early detection of sepsis (KANNEDS) [20]	LSTM^c^	Detect	15,515	8	48 h	0.835	—^d^
Temporal convolutional network [18]	CNN^e^	Detect	40,336	40	Variable	0.91	—
Zhao et al [21]	Light gradient boosting machine	Detect	23,711	25	2,12, 24 h	0.97	—
Li et al [19]	LSTM + CNN	Prediction	40,336	40	11 h	0.964	—
MSTCL^f^ (proposed model)	Multiscale transformer	Online prediction	3766	6	Variable	0.883	0.11

a AUC: area under the curve.

b MAE: mean absolute error.

c LSTM: long short-term memory.

d Not reported.

e CNN: convolutional neural network.

f MSTCL: Multi-Scale Temporal-aware Contrastive Learning.

## Discussion

In this study, we developed a lightweight yet effective framework for real-time sepsis prediction using only 6 commonly available vital signs. Our approach integrates a multiscale transformer-based temporal modeling architecture with a novel temporal-aware contrastive learning strategy, enabling accurate online prediction of sepsis with few input features and significantly fewer samples than most prior studies. This design is motivated by the practical challenge that high-frequency laboratory data and rich clinical notes are often unavailable in many health care settings—particularly in low-resource environments, emergency departments, and wearable-device applications. Our findings demonstrate that sepsis prediction does not necessarily require complex or high-dimensional inputs, as long as the temporal structure of vital sign data is adequately leveraged.

The results from our experiments show that both global and local temporal dependencies play crucial roles in modeling the progression toward sepsis onset. Ablation studies indicate that removing either the global or local attention mechanism substantially degrades predictive performance, underscoring the importance of modeling multiscale dynamics in patient vital signs. This is consistent with prior findings that temporal context and resolution are key factors in accurate time-series modeling for health care applications [22,23]. By combining the 2 scales through residual connections, our model captures both longer-term physiological trends and short-term fluctuations, which are often indicators of clinical deterioration.

Another significant contribution of this work is the introduction of a temporal-aware contrastive learning objective tailored for sepsis trajectory modeling. Unlike standard contrastive learning formulations, which generally rely on static sample augmentations, our framework exploits the natural progression of vital sign sequences by constructing positive, negative, and full-length sequence representations. This hybrid triplet-like structure helps the model better distinguish between subtle temporal variations, especially when labeled data are limited. Similar supervised contrastive approaches have shown promise in related health care domains, such as mortality prediction [24,25] and patient state representation [26,27], and our work further demonstrates their applicability in the context of real-time warning tasks.

Compared with prior studies, our approach requires far fewer training samples. For example, Li et al [19] developed a hybrid CNN-LSTM model that achieved a high AUC (up to 0.96) for sepsis onset prediction but required the full MIMIC dataset and numerous laboratory-based inputs. Other models, such as KANNEDS [20] or light gradient boosting machine–based architectures [21], either relied on fixed-length input windows or used up to 40 types of features, limiting their generalizability and real-time applicability. In contrast, our method achieves comparable performance while being trained on fewer than 10% of the original dataset and using only 6 vital signs, significantly expanding its deployment potential.

A key clinical advantage of our approach is the reliance on only 6 noninvasive vital signs, a strategy aligned with several established models [28,29]. However, we emphasize that the primary innovation of this study is the MSTCL framework itself rather than the specific feature subset. By enforcing contrastive constraints across multiple temporal scales, the framework captures the fine-grained physiological shifts preceding sepsis onset more effectively. This allows our model to bridge the performance gap between limited input and comprehensive input systems, providing a robust tool for high-precision, real-time clinical monitoring.

Our contrastive visualization results further support the model’s ability to learn meaningful representations. As shown in Figure 2, the latent space representations after training display clearer separability between septic and nonseptic patterns, with full-length sequences positioned between the positive and negative extremes. This aligns with clinical intuition and confirms that our contrastive learning framework facilitates more discriminative embedding learning, especially in scenarios with reduced feature sets.

Despite these promising results, there are limitations to our study. First, while the model supports variable-length sequences, extremely short sequences (eg, <4 hours) may not provide enough temporal context for reliable prediction. Although our model is designed to be robust to input length, future work could explore adaptive mechanisms that adjust attention scope based on data sparsity. Second, our evaluation focuses on binary classification of sepsis onset. Extending the framework to estimate time-to-onset as a survival analysis task or to model continuous deterioration risk could offer additional clinical value.

Moreover, our current contrastive design relies on predefined segmentations of sequences into septic and nonseptic windows. While effective, this assumes a clear ground truth onset time in the training phase, which in practice can be noisy or inconsistently recorded across institutions. Future iterations could incorporate soft labels or probabilistic sequence labeling to handle uncertainty in sepsis onset annotations.

From a clinical deployment perspective, the simplicity and efficiency of our model offer distinct advantages. The low computational cost of inference and reliance on readily available vital signs make it suitable for edge deployment on patient monitors or wearable devices. This aligns with broader efforts in ubiquitous health monitoring and early warning systems, where predictive models must operate under resource and latency constraints [30]. The interpretability of the model, particularly via attention visualization or representation clustering, is also a promising direction for integration into clinician-facing decision support tools.

In conclusion, this study presents a novel and efficient framework for online sepsis prediction using only fundamental physiological real-time signals. Through multiscale temporal modeling and contrastive learning, we demonstrate that even with limited data, accurate and timely sepsis detection is achievable. These findings underscore the potential for deploying intelligent monitoring solutions across a wide range of clinical and nonclinical settings, ultimately contributing to better patient outcomes and more efficient resource allocation in critical care.

## Abbreviations

AUC: area under the curve
AUPRC: area under the precision-recall curve
AUROC: area under the receiver operating characteristic curve
CNN: convolutional neural network
ICU: intensive care unit
KANNEDS: kinematics approach with neural networks for early detection of sepsis
LSTM: long short-term memory
MAE: mean absolute error
MSTCL: Multi-Scale Temporal-aware Contrastive Learning
